# Silymarin: Friend or Foe of UV Exposed Keratinocytes?

**DOI:** 10.3390/molecules24091652

**Published:** 2019-04-26

**Authors:** Eszter Fidrus, Zoltán Ujhelyi, Pálma Fehér, Csaba Hegedűs, Eszter Anna Janka, György Paragh, Gábos Vasas, Ildikó Bácskay, Éva Remenyik

**Affiliations:** 1 Department of Dermatology, Faculty of Medicine, University of Debrecen, Nagyerdei krt. 98, 4032 Debrecen, Hungary; hegeduscsaba88@gmail.com (C.H.); janka.eszter.a@gmail.com (E.A.J.); remenyik@med.unideb.hu (É.R.); 2 Department of Pharmaceutical Technology, University of Debrecen, Nagyerdei körút 98, 4032 Debrecen, Hungary; ujhelyi.zoltan@pharm.unideb.hu (Z.U.); feher.palma@pharm.unideb.hu (P.F.); bacskay.ildiko@pharm.unideb.hu (I.B.); 3 Department of Pharmacognosy, University of Debrecen, Nagyerdei körút 98, 4032 Debrecen, Hungary; vasas.gabor@pharm.unideb.hu; 4 Departments of Dermatology and Cell Stress Biology, Roswell Park Comprehensive Cancer Center, Buffalo, NY 14263, USA; gyorgy.paragh@roswellpark.org

**Keywords:** silymarin, antioxidant, UVA radiation, photosensitivity, CPD photolesions

## Abstract

The application of natural plant extracts in UV-protection is popular and intensively studied. Silymarin (from *Silibum marianum*), a naturally occurring polyphenol, has recently received attention due to its antioxidant, anti-inflammatory and anti-apoptotic effects. However, its role in the UV-mediated keratinocyte cell response is still controversial. In this study, we investigated the effects of *Silibum marianum* extracts with different origins and formulations on UVA-exposed HaCaT keratinocytes in vitro. Our results show, that silymarin treatment caused an inverse dose-dependent photosensitivity relationship (at higher doses, a decrease in cell viability and ROS production) after UVA exposure. The attenuation of the UVA-induced ROS generation after silymarin treatment was also observed. Moreover, silymarin pre-treatment increased the cyclobutane pyrimidine dimer photolesions in keratinocytes after UVA exposure. These results indicated the dual role of silymarin in UVA-exposed keratinocytes. It scavenges ROS but still induces phototoxicity. Based on our results dermatological applications of silymarin and related compounds should be considered very carefully.

## 1. Introduction

More than 90% of solar ultraviolet (UV) radiation reaching the Earth’s surface falls within the 315 nm to 400 nm (UVA) wavelength range [1]. Although the shorter-wavelength (320–290 nm) UVB is considered to be the main carcinogenic component of the solar UV spectrum, the impact of UVA radiation on sunburn, photoaging and carcinogenesis of the human skin cannot be underestimated [1,2,3].

Accumulating evidence suggests that the main cause of the UVA-induced cytotoxicity and mutagenesis can be attributed to the production of intracellular reactive oxygen species (ROS) [1,2,3,4], which are generated by the direct excitation of endogenous chromophore molecules including tryptophan, porphyrins, melanin [4]. These reactive oxygen species can interact with intracellular macromolecules leading to lipid peroxidation [5], protein oxidation [6] and DNA base modifications [7,8,9].

7,8-Ddihydro-8-oxoguanine (8-oxoG) lesions are the most common and intensively studied UVA-induced DNA alteration, which are generated by singlet oxygen molecules interacting with guanine bases and causing G-T transversion during DNA replication [1,10]. However, recent studies have found that a significant amount of cyclobutane pyrimidine dimer (CPD) photolesions are also produced by UVA radiation [9,11,12]. CPDs are formed from two covalently linked, adjacent pyrimidine bases, which can cause replication failures, single-base mismatches and DNA double strand breaks [13], leading to cell-cycle arrest [14], apoptosis [15] and the accumulation of DNA mutations [16,17]. Although UVB radiation is predominantly responsible for CPD formation, several studies show that UVA can also substantially contribute to the generation of these photolesions by a different mechanism. While UVB induces CPD formation via direct absorption of the UVB photons on the DNA [11], UVA radiation produces CPDs indirectly by triplet energy transfer from a recently unknown endogenous chromophore excited by UVA photons [11,12]. Nevertheless, the exact mechanism of UVA-induced CPD formation remains unclear. 

A wide range of naturally occurring phytochemicals are intensively studied for their ROS scavenging ability to prevent the deleterious effect of UVA on human skin [18,19,20,21,22]. Silymarin is a flavonoid complex extracted from the seeds of milk thistle (*Silibum marianum*). It contains numerous bioactive components (e.g., silibinin, silychristin, silydianine and taxifolin), many of which have shown strong antioxidant [23,24], anti-inflammatory [24,25] and immunomodulatory [26] potential. Through these mechanisms, silymarin was found to protect against the UVA-induced apoptosis and carcinogenesis [27,28]. UVB-protecting properties of the components were also shown [29,30,31].

Nonetheless, some studies showed that silymarin enhances the UVA-induced cell death and thus serves as a photosensitizer [32,33]. Katiyar et al. found that silymarin induced apoptosis via modulation of the p53 and NFκB pathways [34], but the exact mode of action of silymarin is still unclear. Photosensitization is a widely known phenomenon in the field of photobiology, when endo-or exogenous chromophore molecules absorb the energy of the irradiating light with a specific wavelength [4,35]. The excited chromophores interact with other molecules, such as cellular lipids, proteins or nucleic acids. In Type I. photosensitization reaction there is a direct electron transfer between the photosensitizer and the substrate molecules, which can act as free radicals and produce oxidized products in the presence of molecular oxygen. These oxidized molecules can react further with other substrates and induce changes in their structure and function [1,3,35]. In Type II. photosensitization reaction there is an energy transfer from the excited photosensitizer to molecular oxygen, and the generated singlet oxygen interacts with biological substrates [1,3,35]. Oxygen-independent, type III and IV. photosensitization reactions also exist, but their mechanisms of action are poorly understood [35].

In summary, the effects of silymarin on the cellular UV-damage is fairly controversial. Studies showing silymarin’s positive and negative effects on UV-induced cytotoxicity also exist. The molecular mechanisms behind the potential photosensitizer effect of silymarin are little understood. 

To understand the photobiological effect of silymarin, we investigated the effects of three silymarin compounds with different origin and composition on UVA-irradiated HaCaT keratinocyte cell line. We found that silymarin had dual effect on UVA-irradiated keratinocytes: it enhanced the UVA-induced cell death but decreased the intracellular ROS level after a high-dose of UVA irradiation. Furthermore, we found that silymarin increased the amount of CPD photolesions in the cells after UVA-exposure. Our results suggest that silymarin shows versatile effects on UVA-exposed HaCaT keratinocytes. Therefore, dermatological applications of silymarin should be considered very carefully due to its possible adverse impacts.

## 2. Results

### 2.1. Silymarin Pre-Treatment Enhances the UVA-Induced Cytotoxicity

To determine the effects of UVA radiation and silymarin treatment on cell viability, HaCaT keratinocytes were exposed to a single-dose of 10 or 20 J/cm^2^ UVA or left sham-irradiated. Silymarin treatments were performed for 30 min immediately before UVA irradiation in different doses and composition. We applied:

(1) A silymarin extract (Sigma-Aldrich, St. Louis, MO, USA) dissolved in ethanol.
(2) A commercially available silymarin compound (Silegon, Teva Pharmaceutical Industries Ltd., Petach Tikva, Israel).
(3) Four different topical formulations of silymarin containing 250 µg/mL herbal extract dissolved in Transcutol HP (TC) and different sucrose-esters as penetration enhancers (see Table 1 at Materials for details). We have previously shown the efficacy of these enhancers on cell and skin penetration [36].

**Figure 1:**
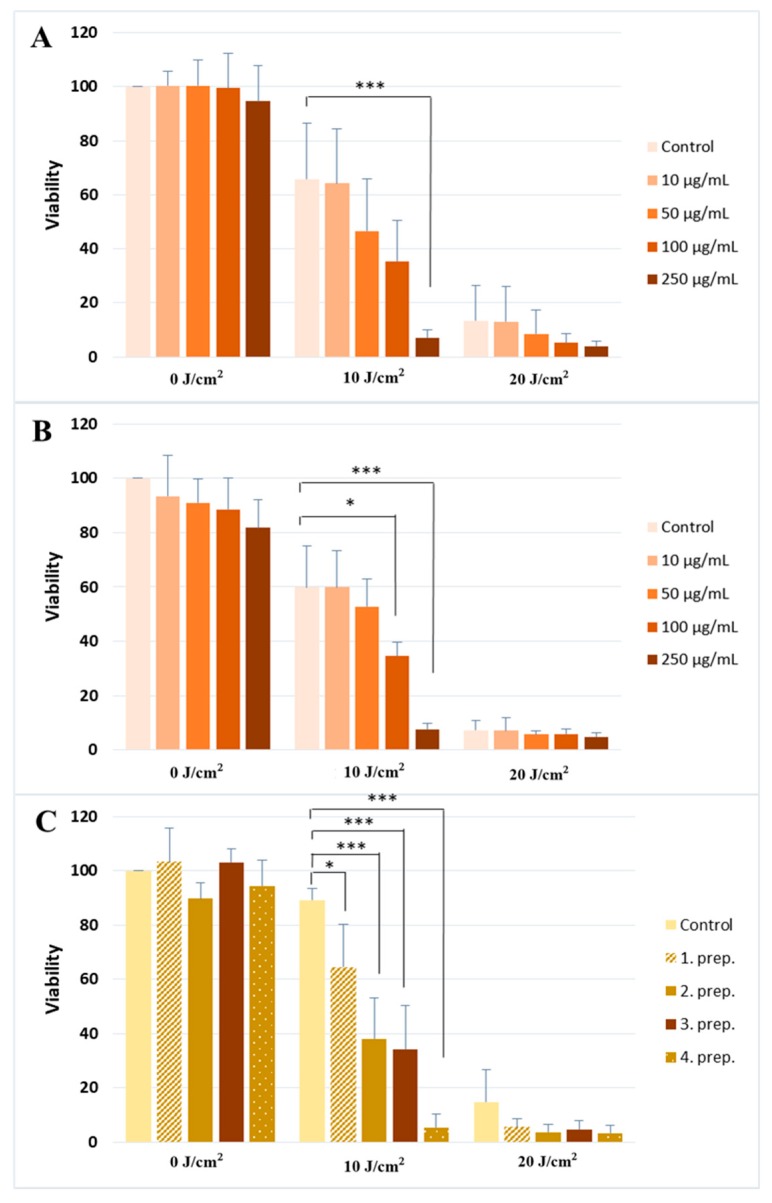
UVA photosensitizing effect of different silymarin preparations. Cell viability after UVA exposure and pretreatment with (**A**) silymarin mixture (Sigma) (**B**) a commercially available silymarin compound (Silegon, Teva), and (**C**) 250 μg/mL silymarin dissolved in four different penetration-enhancers. The results are the mean of four independent experiments. Error bars represent SD; * and *** indicate statistically significant difference at *p* < 0.05 and *p* < 0.001, respectively.

Twenty four h after the UV-exposure, the relative cytotoxic effects of the treatments were measured by 3-[4–dimethylthiazol-2-yl]-2,5-diphenyltetrazolium bromide (MTT) assay. We found that UVA radiation caused a dose-dependent decrease in cell viability. The UVA-induced cytotoxicity was strongly increased by silymarin pre-treatment. Decrease in cell viability was dependent on silymarin concentration: 10 J/cm^2^ UVA resulted in 30–40% apoptosis, whereas 50 and 100 μg/mL silymarin dissolved in ethanol augmented cell death of UVA-irradiated cells to 40–60%. More than 90% of the UV-exposed cells were dead after 250 μg/mL silymarin pre-treatment. Silymarin and Silegon had similar effects on cell survival (Figure 1A,B, respectively). Silymarin dissolved in penetration enhancers showed the same effect, the extent of phototoxicity varied based on the composition of the different formulations (Figure 1C).

**Figure 2:**
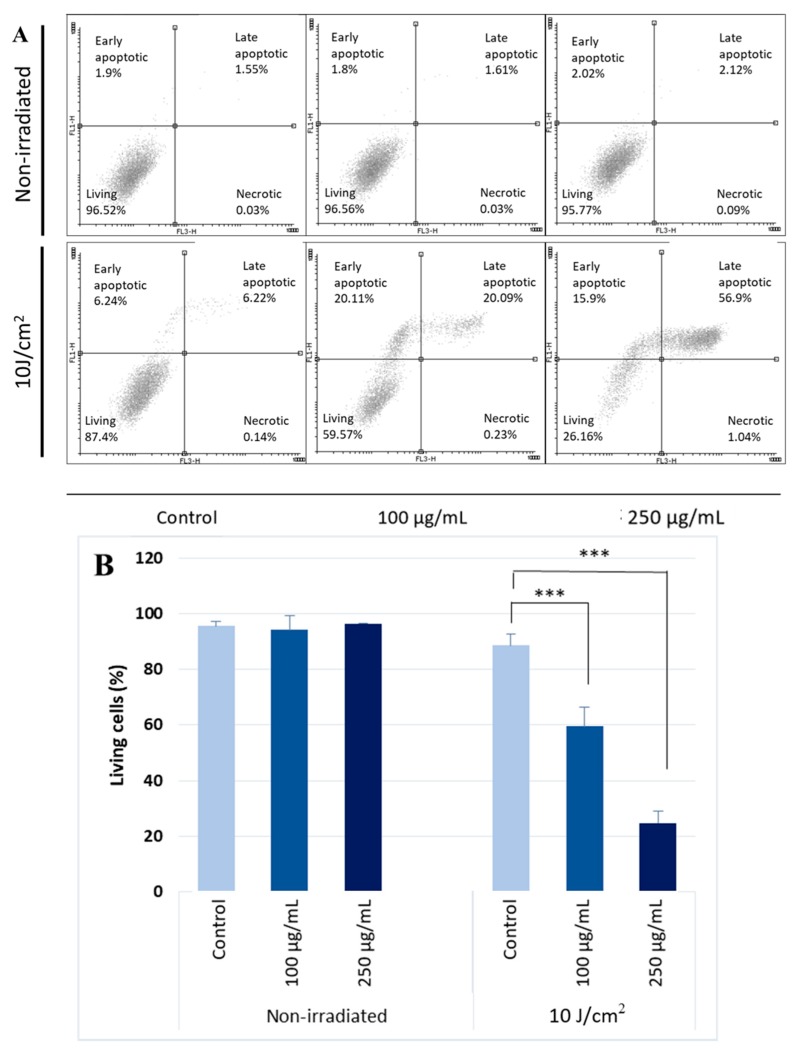
UVA photosensitizing effect of silymarin. (**A**) Effect of 10 J/cm^2^ UVA radiation and silymarin + UVA co-treatment was measured by Annexin V and propidium iodide dual staining. (**B**) Live percentage as a mean of three independent experiments after silymarin treatment. Error bars represent SD; *** indicates statistically significant difference at *p* < 0.001.

The results of the MTT assays were confirmed with Annexin V and propidium iodide dual staining followed by flow cytometry. We chose the two most cytotoxic concentrations of silymarin (Sigma) for assessing apoptosis. The UVA-dose was 10 J/cm^2^. Silymarin also showed marked photosensitizing potential with this assay, while 10 J/cm^2^ UVA irradiation alone caused a moderate decrease in cell viability (Figure 2).

### 2.2. Silymarin Treatment Reduces Intracellular ROS Production after UVA Irradiation

According to several studies [23,24,27,28], silymarin may have a strong antioxidant potential. To test the influence of silymarin compounds on ROS production after UVA exposure, we stained cells with dihydroethidium (DHE) and fluorescence intensity was analyzed by flow cytometry.

**Figure 3:**
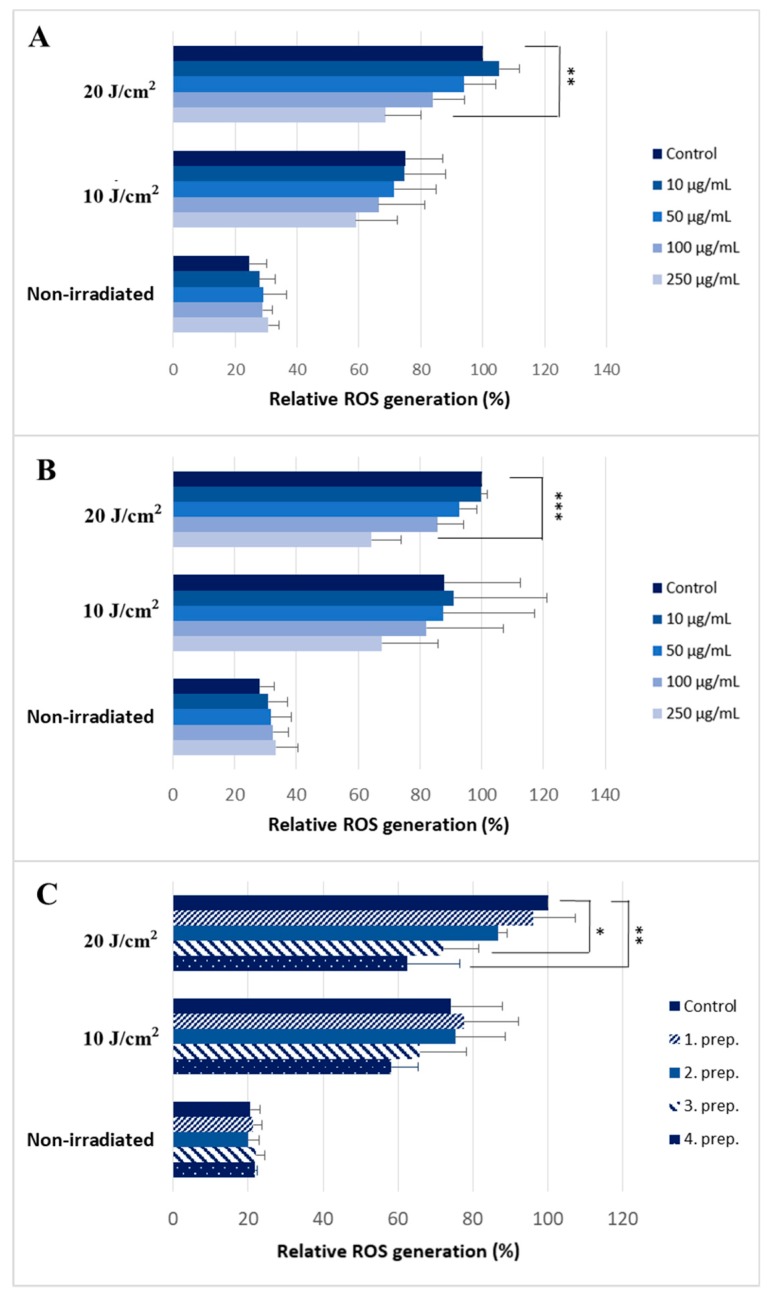
Antioxidant effect of silymarin after UVA-irradiation. Cellular ROS levels after UVA exposure following pretreatment with (**A**) silymarin mixture (Sigma) (**B**) a commercially available silymarin compound (Silegon, Teva), and (**C**) 250 μg/mL silymarin dissolved in four different penetration-enhancers. The bars represent the mean of three independent experiments. Error bars depict SD; *, ** and *** indicate statistically significant difference at *p* < 0.05, *p* < 0.01 and *p* < 0.001, respectively.

We found that silymarin pre-treatment reduced ROS production of skin keratinocytes after high-dose (20 J/cm^2^) UVA irradiation in a dose-dependent manner. 250 μg/mL silymarin caused a 30% decrease in DHE intensity (Figure 3A,B).

Two silymarin compounds supplemented with penetration enhancers (compositions 3 and 4) had the same effect. The compound with the greatest ROS scavenger activity reduced ROS level by 40% (Figure 3C). Earlier this composition was found to be the most phototoxic, too. Although a trend towards decreased ROS was observed the antioxidant activity of the silymarin compounds after low-dose (10 J/cm^2^) of UVA radiation was not significant. Baseline ROS levels were not affected by silymarin treatment.

### 2.3. Silymarin Pre-Treatment Enhances the UVA-Induced CPD-Generation

**Figure 4:**
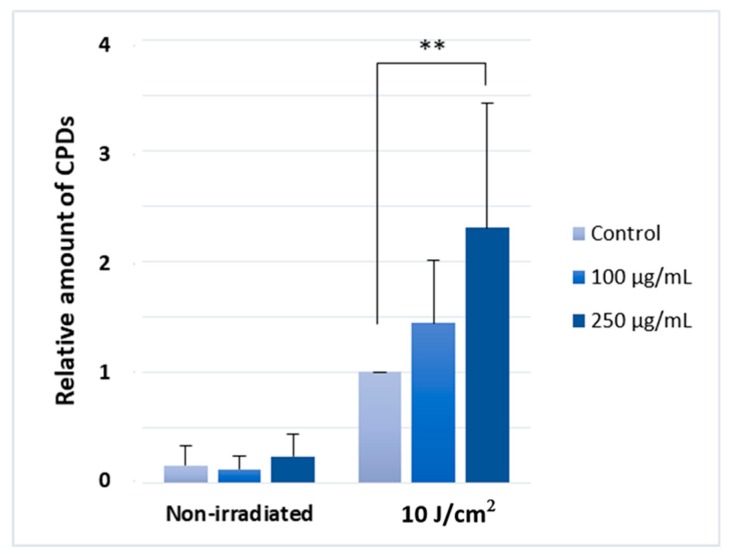
The relative amount of CPD photolesions after UVA irradiation and silymarin co-treatment. The results are the mean of six independent experiments. Error bars represent SD; ** indicates statistically significant difference at *p* < 0.01 mL and please change 0,5 1,5 2,5 as 0.5 1.5 2.5 in the Y-axis.

The mechanism underlying silymarin induced phototoxicity is not well understood. Other photosensitizing chemicals were found to contribute to CPD formation after UV radiation [37,38]. To test whether CPD induction may contribute to silymarin phototoxicity, we measured the UV-induced CPD formation in silymarin treated keratinocytes after UVA-exposure. We found, that UVA and silymarin co-treated cells showed a significantly higher amount of CPDs compared to the UVA-exposed cells (10 J/ cm^2^). As earlier, we used the two most cytotoxic concentrations of the silymarin (Sigma) (Figure 4). 

## 3. Discussion

Naturally occurring plant extracts are often used in scientific investigations aiming to prevent the detrimental biologic effects of UV-radiation on the human skin [18,19,20,21,22]. Silymarin is a mixture of different bioactive flavonoid components derived from the seeds of milk thistle (*Silibum marianum*). The protective effects of silymarin and its components on UVA-damage of the human skin cells were demonstrated in several scientific works. This positive effect is mostly attributed to the antioxidant properties of silymarin, thereby the reduction of the UVA-induced ROS production [23,24,27]. Nonetheless, other studies found that silymarin acts as a strong photosensitizer by significantly reducing the cell survival after UVA irradiation. The exact mechanism of this photosensitivity reaction is mostly unknown [32,33]. Based on these conflicting results, currently the use of silymarin in UV-protection is controversial.

In the present study we demonstrated that the antioxidant and phototoxic properties of silymarin may appear simultaneously. Silymarin enhanced the UVA-induced cell death in HaCaT keratinocytes but reduced intracellular ROS level, suggesting that it acts as a photosensitizer and a ROS scavenger at the same time. We hypothesized that the reason of this dual effect is derived from the variety of the components. Silymarin is a mixture containing a large number of flavonolignans with different structures [39], which likely differ in their UVA interactions and post-UVA biological properties. Some individual silymarin components were defined as photosensitizers, although their role is still contradictory [32,33,34]. The ultimate biological effect of silymarin may depend on the concentration of the components in the mixture, the penetration of the components across the cell membrane and other experimental circumstances. Dhanalakshmi et al. found that silibinin, a main component of silymarin can enhance or decrease the UVB-induced cytotoxicity depending on the UVB-dose [40] further underlining the complexity of silymarin UV-phototoxicity. Silymarin components were not investigated in this study individually.

We found, that silymarin pre-treatment increased the detectable amount of potentially highly mutagenic CPD photolesions after UVA irradiation. Kunisada et al. and Robinson et al. found a similar increase in UVA-induced CPDs after hydrochlorothiazide (HCT) [37] and carprofen pre-treatment [38], however, phytoflavonoid-mediated UVA-dependent CPD induction has not previously been described. Further investigations are needed to understand the exact mechanism of CPD increase. As UVA induces limited CPDs in cell culture in the absence of melanin [41], silymarin treatment more likely enhances CPD-induction via an unknown mechanism that suppresses DNA repair. The underlying CPD generation likely contributes to the observed photosensitizing effect of the silymarin.

In conclusion, the utility of currently available silymarin mixtures in UV-protection is questionable. Future studies need to examine the effects of individual silymarin components for identification of potential mutagenic, cytotoxic and possibly photoprotective molecules. Our results suggest that dermatological applications of natural flavonoids require careful testing and thoughtful assessment of their potential UV interaction to limit and avoid possible adverse effects.

## 4. Materials and Methods

### 4.1. Cell Culture

Human immortalized keratinocyte-derived (HaCaT) cell line was cultured in T75 flasks as previously described [42]. An established HaCaT cell line was maintained in high glucose DMEM with L-209 glutamine and sodium pyruvate (Biosera, Nuaille, France) supplemented with 10% fetal bovine serum (Biosera) and 0.5% antibiotic/antimycotic (penicillin-streptomycin-amphotericin B solution, Biosera) and maintained at 37 °C with a 5% CO_2_ atmosphere.

### 4.2. Preparation of Silymarin Formulations

#### 4.2.1. Materials

We applied three silymarin sources at our experiments. The first was a gift from Ákos Kuki (Department of Applied Chemistry, University of Debrecen, Debrecen, Hungary). Silymarin powder from *Silibum marianum* seeds was prepared according to Kahol et al. [43]. The silymarin powder did not contain any solvent residue. The same bioactive flavonolignans were determined as in the standards with the help of HPLC-MS method. The exact composition of the silymarin powder was published in the previous work of Kuki et al. [44]. The second source of silymarin was silymarin flavonolignans ordered from Sigma-Aldrich (St. (Louis, MO, USA). The third origin of silymarin was Silegon (Teva Pharmaceutical Industries Ltd., Petach Tikva, Israel) a commercially available dragée containing silymarin.

Transcutol was a kind gift from Gattefossé (Saint-Priest, France). Sucrose esters (SP50, SP70) were kind gifts from Sisterna (Roosendaal, The Netherlands) Propylene glycol was supplied by Hungaropharma Ltd., (Budapest, Hungary). Human keratinocyte (HaCaT) cells were obtained from Cell Lines Service (CLS, Heidelberg, Germany).

#### 4.2.2. Preparation of Compositions Containing Silymarin Powder

For all compositions 1–4 (see Table 1 for details) the propylene glycol and the emulsifying agent (sucrose ester SP50 or SP70) was dissolved in the cell culture media at 37 °C and then cooled down to 25 °C. Finally silymarin powder was added to the compositions. For compositions 3 and 4 silymarin powder was previously dissolved in Transcutol.

Silymarin flavonolignans and pulverized Silegon tablets were dissolved in 96% ethanol and diluted in complete DMEM to a final concentration of 10, 50, 100 or 250 μg/mL.

### 4.3. Silymarin Treatments

HaCaT cells were seeded into 96-well (for MTT assay) or 24-well plates (for other measurements) and grown near to confluence. Cells were treated with 10–250 μg/mL silymarin (Sigma or Teva), the final concentration of ethanol in the culture medium did not exceed 0.5%. The extracts dissolved in the penetration enhancers were used at a concentration of 250 μg/mL for cell treatment. HaCaT keratinocytes were incubated 30 min with the silymarin extracts at 37 °C prior to UV irradiation.

### 4.4. UVA Irradiation

Immediately after silymarin treatment, cells were washed twice with DPBS (Biosera) and irradiated with a single-dose of 10 or 20 J/cm^2^ UVA (PUVA 800, H. Waldmann GmbH & Co. KG, Villingen-Schwenningen, Germany) under a thin layer of PBS complemented with d-glucose (Duchefa Biochemie B.V. Haarlem, The Netherlands). Proper dosage of UVA was determined by a UVX Digital Radiometer (UVP Inc., San Gabriel, CA, USA). UVA irradiation was performed on ice to avoid heat shock. Control cells were also placed on ice during the irradiation time, but covered to be protected from UVA. After UVA-exposure, complete DMEM (without silymarin) was added. 

### 4.5. MTT (3-[4–dimethylthiazol-2-yl]-2,5 diphenyl tetrazolium bromide) Assay

The viability of HaCaT cells were measured by MTT (3-[4–dimethylthiazol-2-yl]-2,5 diphenyl tetrazolium bromide) assay 24 h after UVA irradiation. Cells were washed with PBS, then 100 μL/well DMEM without phenol red (HyClone; GE Healthcare Life Sciences, Logan, UT, USA) containing 200 μg/mL MTT (ThermoFisher Scientific, Waltham, MA, USA) were added. Cells were incubated for 3 h at 37 °C in 5% CO_2_ atmosphere. At the end of the incubation period, media was removed and 0.04 M HCl in isopropanol was added to solubilize the formazan crystals. Absorbance was measured at 590 nm with background subtraction at 620 nm by an Epoch Microplate Spectrophotometer (BioTek, Winooski, VT, USA). 

### 4.6. Annexin V/Propidium Iodide Staining (Apoptosis Assay)

After 24 h post-UVA irradiation, detached cells were collected by aspirating the media and adherent cells were collected by 1x trypsin-EDTA solution and added to the supernatant. 0.1 μL/mL Alexa Fluor 488 annexin V and 1 μg/mL propidium iodine (PI) were dissolved in 1X annexin-binding buffer (Alexa Fluor 488 Annexin V/Dead Cell Apoptosis Kit, ThermoFisher, Waltham, MA, USA), and 100 μL working solution were added to each sample. Stained cells were analyzed by FACSCalibur flow cytometer (BD Biosciences, San Jose, CA, USA) using CellQuestPro software (BD Biosciences) and fluorescence intensity was measured in FL1 (for Annexin V) and FL3 (for PI) channel, respectively.

### 4.7. ROS (Reactive Oxygen Species) Production Measurements

Intracellular ROS detection was carried out by dihydroethidium (DHE) staining followed by flow cytometry analysis. Immediately after UVA irradiation, cells were washed with PBS and 200 nM DHE (ThermoFisher) in PBS was added to each well. Cells were incubated 30 min at 37 °C and trypsinized. 500 μL complete DMEM was added to neutralize the trypsin-EDTA solution (Biosera). Samples were analyzed by a FACSCalibur flow cytometer (BD Biosciences) using CellQuestPro software (BD Biosciences), and fluorescent signal was measured in FL3.

### 4.8. Enzyme-Linked Immunosorbent Assay (ELISA)

CPD-specific ELISA was established by Boros et al. in our previous work [45]. 24 h after the UVA irradiation, genomic DNA was extracted by an Invitrogen™ PureLink™ Genomic DNA Mini Kit (ThermoFisher), according to the manufacturer’s instruction. Flat-bottom 96-well plates were coated with 0.003% protamine-sulfate (Sigma-Aldrich) and incubated at 37 °C by drying completely. DNA was denaturated at 100 °C for 10 min, then immediately chilled on ice for 15 min. Denaturated DNA was distributed to wells in triplicate (15 ng DNA to each well), and incubated at 37 °C overnight. Plates were washed with PBS containing 0.05% Tween-20 (VWR, Radnor, PA, USA) and incubated with 150 μL/well 5% FBS at 37 °C for 30 min to prevent non-specific antibody binding. Plates were washed three times with PBS-T, then anti-CPD monoclonal antibody (clone TDM-2, dilution 1:1500, Cosmo Bio Co., Ltd., Tokyo, Japan) was added to each well and plates were incubated at 37 °C for 60 min. Plates were washed three times, and incubated with HRP-conjμgated anti-mouse IgG secondary antibody (dilution 1:3000, Bio-Rad, Hercules, CA, USA) at 37 °C for 30 min. Plates were washed three times with PBS-T and once with 150 μL/well citrate-phosphate buffer (0.51% C_6_H_8_O_7_.H_2_O (Sigma) and 0.73% Na_2_HPO_4_ (Sigma) in distilled water; pH 5.0), then substrate solution (0.04% *o*-phenylenediamine (Sigma-Aldrich) and 0.006% H_2_O_2_ in citrate-phosphate buffer) was added to each well and incubated until the appropriate color intensity appear. To stop the enzyme reaction, 50 μL/well 2N H_2_SO_4_ was added. Absorbance was measured at 492 nm using an Epoch Microplate Spectrophotometer (BioTek). 

### 4.9. Statistical Analysis

The distribution of data was analyzed by Kolmogorov–Smirnov test. If the distribution was normal, we used ANOVA followed by Dunnett’s post-hoc test to determine significance between the control and different treatment groups. In case the data did not show a normal distribution, Kruskal-Wallis test were applied complemented by Dunn’s post-hoc test. The significance level was set at 0.05.

## Figures and Tables

**Table 1:** Preparation of compositions containing silymarin powder with penetration enhancers.

	Preparations			
Ingredients (g)	1	2	3	4
**Silymarin powder**	0.25	0.25	0.25	0.25
**Transcutol**	**----**	**-----**	**0.71**	**0.71**
**Sucrose ester SP50**	**0.15**	**----**	**0.15**	**---**
**Sucrose ester SP70**	**---**	**0.15**	**---**	**0.15**
**Propylene Glycol**	0.25	0.25	0.25	0.25
**Cell culture media**	ad 100	ad 100	ad 100	ad 100
